# Effect of Interpectoral-Pectoserratus Plane (PECS II) Block on Recovery Room Discharge Time in Breast Cancer Surgery

**DOI:** 10.3390/medicina60010041

**Published:** 2023-12-25

**Authors:** Laima Malachauskiené, Rajesh Prabhakar Bhavsar, Jacob Waldemar, Thomas Strøm

**Affiliations:** 1 Department of Anesthesia and Critical Care Medicine, South Jutland Regional Hospital, Kresten Philipsens Vej 15, DK-6200 Aabenraa, Denmark; laima.malachauskiene@rsyd.dk (L.M.); thomas.stroem@rsyd.dk (T.S.); 2 Odense Medical College, Odense University, Campusvej 55, DK-5230 Odense, Denmark; jacob.waldemar@rsyd.dk

**Keywords:** breast cancer, mastectomy, PECS block

## Abstract

*Background and Objectives*: The increase in the incidence and diagnosis rate of breast cancer demands the optimization of resources. The aim of this study was to assess whether the supplementation of the interpectoral-pectoserratus plane block (PECS II) reduces surgery and post-anesthesia care unit (PACU) time in patients undergoing breast cancer surgery. *Materials and methods*: This was a retrospective data-analysis study. In 2016, PECS II block was introduced as a supplement to general anesthesia for all mastectomies with or without axillary resections in South Jutland regional hospital, Denmark. The perioperative data of patients operated 3 years before and 3 years after 2016 was retrieved through the Danish anesthesia database and patient journals and systematically analyzed. Female patients aged over 18 years, with no use of muscle relaxant, intubation, and inhalation agents, were included. The eligible data was organized into two groups, i.e., Block and Control, where the Block group received PECS II Block, while the Control group received only general anesthesia. Parameters such as surgery time, anesthesia time, PACU time, opioid consumption, and the incidence of postoperative nausea and vomiting (PONV) in PACU were retrieved and statistically analyzed. *Results*: A total of 172 patients out of 358 patients met eligibility criteria. After applying exclusion criteria, 65 patients were filtered out. A total of 107 patients, 51 from the Block and 56 from the Control group, were eligible for the final analysis. The patients were comparable in demographic parameters. The median surgery time was significantly less in the Block group (78 min (60–99)) in comparison to the Control group (98.5 min (77.5–139.5) *p* < 0.0045). Consequently, the median anesthesia time was also shorter in the Block group (140 min (115–166)) vs. the Control group (160 min (131.5 to 188), *p* < 0.0026). Patients from the Block group had significantly lower intraoperative fentanyl consumption (60 µg (30–100)) as compared with the Control group (132.5 µg (80–232.5), *p* < 0.0001). The total opioid consumption during the entire procedure (converted to morphine) was significantly lower in the Block group (16.37 mg (8–23.6)) as compared with the Control group (31.17 mg (16–46.5), *p* < 0.0001). No statistically significant difference was found in the PACU time, incidences of PONV, and postoperative pain. *Conclusions*: The interpectoral-pectoserratus plane (PECS II) block supplementation reduces surgery time, anesthesia time, and opioid consumption but not PACU time during breast cancer surgery.

## 1. Introduction

Breast cancer is the second-most common neoplastic disease among women, where over 2.26 million new cases were observed globally in 2020 [1]. In Denmark, more than 4800 women are diagnosed with breast cancer each year. Due to this increased incidence, a national screening initiative has been implemented in recent years. A considerable rise in the detection of new breast cancer incidences is expected after the national breast cancer surveillance program [2]. There are various single or combination therapeutic approaches for the treatment of breast cancer, such as hormonal therapy, chemotherapy radiotherapy, and surgery. Among the various surgery options, mastectomy with axillary resection is one of the major curative treatments for breast cancer [3]. To accommodate the additional flow of patients, surgical units, i.e., operation theaters (OTs), post-anesthesia care units (PACUs), and surgical wards, need continuous adjustments in capacity. As a logical solution, an OT must be utilized for longer durations each day, or the number of OTs allotted for breast surgical patients should be increased [4,5]. Both these interventions may increase the demand for resources, which may be challenging with the present workforce situation in Western countries, including Denmark [6]. However, by decreasing the duration required for the entire procedure, more patients may be accommodated during the same timeslot without the need for more staff. Additionally, a recent meta-analysis showed that prolonged operative time is associated with an increase in the risk of adverse complications. Therefore, decreased operative times should be a universal goal for surgeons, hospitals, and policy makers [7].

As the duration of the occupancy of an OT is defined by surgery, anesthesia time, and post-operative nausea and vomiting (PONV), and pain define the duration of stay in the PACU [8], the efforts towards acceleration of the flow in the operation unit were naturally focused on optimization of surgical procedure time, anesthesia, and PACU time.

Surgical procedure time depends on multiple factors, including patient comorbidities such as uncontrolled hypertension, anatomical challenges, anticoagulation status, and the skills and experience of the surgical team. Unless the surgical technique is changed entirely, e.g., from an invasive to an endoscopic approach, surgery times usually remain relatively consistent in an adequately experienced surgical team. Therefore, an improvement in the surgical procedure time that can achieve a logistical advantage in patient flow acceleration may appear challenging.

On the other hand, anesthesia has the potential to control the entire duration of stay of the patients in the surgical unit, as it influences multiple factors, such as pain, nausea, vomiting, respiratory depression, etc., which decide the major postoperative course of events.

The most important part of anesthetic management, which has a considerable influence on the course of perioperative events, is analgesia, especially opioids, which are integral of perioperative anesthetic management. However, along with potent analgesic properties, the majority of opioid preparations have side effects, such as PONV and respiratory depression, which may prolong the stay in PACU. In previous studies it has been observed that these side effects are dose-related [9]. Various approaches to optimize the dose of opioids enough to avoid postoperative complications have been investigated [9]. Amongst these methods, the supplementation of regional anesthesia for the reduction of the intraoperative consumption of opioids has been increasingly considered [10]. As the surgical field for breast operations involves sensory innervation predominantly from intercostal nerves, various approaches to block the nociception from the surgery through local anesthetic infiltration at various levels have been investigated [11]. A review of earlier observations revealed that focused pectoralis nerve blocks (PECSs), where the sensory nerves supplying only the breast region are blocked, may be considered as a supplement during breast cancer surgery [11,12,13].

The novel PECS block technique was first described by Blanco et al. [14] in 2011. The PECS I block is a high-volume interfascial block between the pectoralis major muscle and pectoralis minor muscle, targeting the lateral pectoral nerves. Further, in 2012, the same research group described a second version of the PECS block called modified PECS block or PECS II block [15]. The PECS II targets the interfascial plane between the pectoralis major muscle and the pectoralis minor muscle, as does the PECS I, and also targets the interfascial plane between the pectoralis minor muscle and the serratus anterior muscle, aiming to block intercostal nerves three to six, the intercostobrachial nerve, and the long thoracic nerves, all of which are necessary for axillary node dissection. A new name, i.e., interpectoral-pectoserratus plane block, has been recommended through the ASRA–ESRA Delphi consensus study for the PECS II block [16].

With this knowledge, since 2016, every patient undergoing breast cancer surgery at Southern Jutland Regional Hospital has received interpectoral-pectoserratus plane (PECS II) block as a supplement to general anesthesia.

Previous investigations on the PECS II block were predominantly focused on PONV and postoperative pain, and revealed varied outcomes. Very few studies have investigated its impact on the duration of stay in the PACU, and particularly in the rest of the surgical unit, along with its further benefits in increasing patient flow.

Therefore, through the retrospective analysis of patient records, we intended to assess whether the changed anesthetic strategy offers advantages in postoperative pain and PONV. Further, the focus was to evaluate whether PECS block offers benefits for parameters such as anesthesia and PACU time, which are relevant for logistic management and resource optimization.

## 2. Materials and Methods

Permissions to access the journals of patients operated for breast surgery were obtained from the regional review board, the Danish Anesthesia Database (DAD) regulatory authority, and the national data security agency. The perioperative journals of patients undergoing surgery between January 2013 to December 2015 (3 years before the implementation of the PECS block as part of standard anesthetic management) were treated as the Control group. Similarly, journals of patients undergoing surgery between January 2016 to December 2018 (3 years after the implementation of the PECS block), were treated as the Block group.

### 2.1. Study Population

The study included female patients of ≥18 years of age who were undergoing mastectomy with or without axillary resection. Patients were identified with the help of procedure codes for the specified surgery through the Danish National Anesthesia database (DAD). Patients who received intraoperative muscle relaxants, intubation, and those who received inhalational agents, were excluded from the study. Similarly, patients with missing data and patients who underwent surgery after 2016 and did not receive the block were not included in the analysis.

The majority of intra-operative and postoperative data could be retrieved through the perioperative anesthesia chart. Further information such as comorbidity factors, preoperative medical treatment, management in the wards, discharge, etc. was accessed through patients’ electronic journals.

### 2.2. Clinical Outcomes

The primary outcome was the duration of stay in the PACU. Secondary outcomes included surgery time, anesthesia time, entire procedure time, the intraoperative use of opioids, postoperative and total opioid consumption, pain at arrival in the PACU, and the incidence of PONV.

### 2.3. Anesthesia/Analgesia Technique

The anesthesia technique followed institutional recommended guidelines. The anesthetic induction was carried out using intravenous Fentanyl (1.5–2.5 μg/kg) or Sufentanil (0.2–0.3 μg/kg) as an analgesic, followed by Propofol (2.0–2.5 mg/kg) as a hypnotic agent. After routine pre-oxygenation with 100% oxygen for 3 min, the airway was secured with a laryngeal mask airway (LMA). The anesthesia was maintained by using the total intravenous anesthesia (TIVA) technique through infusions of Propofol (5–7 mg/kg/h) and Remifentanil (20–40 μg/kg/h). The supplementation of additional analgesics was at the discretion of the attending anesthesiologist and was typically based on hemodynamic indicators and previous experience. Hemodynamic management and fluid supplementation followed standard practice and was uniform during the entire study period. The surgical technique was uniform throughout the investigation period, and no additional local anesthetic was used as infiltration by the surgeon.

### 2.4. PECS II Block Technique

The ultrasound-guided interpectoral-pectoserratus plane (PECS II) block was performed following institutional recommended guidelines. The block procedure was conducted immediately after the anesthesia induction. A local anesthetic infiltration was performed at the levels of the third and fourth ribs at the mid-axillary line. The interpectoral block was performed by introducing the needle into the plane from medial to lateral. A total of 20 mL of 0.2% Ropivacaine was injected in the interfascial plane between pectoralis minor and pectoralis major muscles. The pectoserratus block was performed by injecting 20 mL of 0.2% Ropivacaine between the pectoralis minor and serratus anterior muscles. Perioperative monitoring and postoperative management were as per the standard protocol for all patients.

The data were organized and made suitable for statistical analysis; where intraoperative opioids were converted to micrograms of Fentanyl for intraoperative comparison and Morphine for perioperative comparison. Postoperative opioids were converted to mg of Morphine for comparison. The used conversion factor was as advised by the National Drug and Alcohol Research Centre, University of NSW “https://ndarc.med.unsw.edu.au/sites/default/files/ndarc/resources/TR.329.pdf (accessed on 24 January 2023)”.

### 2.5. Statistics

Patient demographics and clinical characteristics were summarized for the two study groups using descriptive statistics and tested for normality. Categorical variables were compared using the Chi^2^ test, and continuous variables were compared using the Mann–Whitney test, one-way ANOVA, or Kruskal–Wallis, as appropriate. All calculations were conducted using STATA 17 (Stata Corp. 2021. Stata Statistical Software: Release 17. Stata Corp LLC: College Station, TX, USA).

## 3. Results

**Figure 1:**
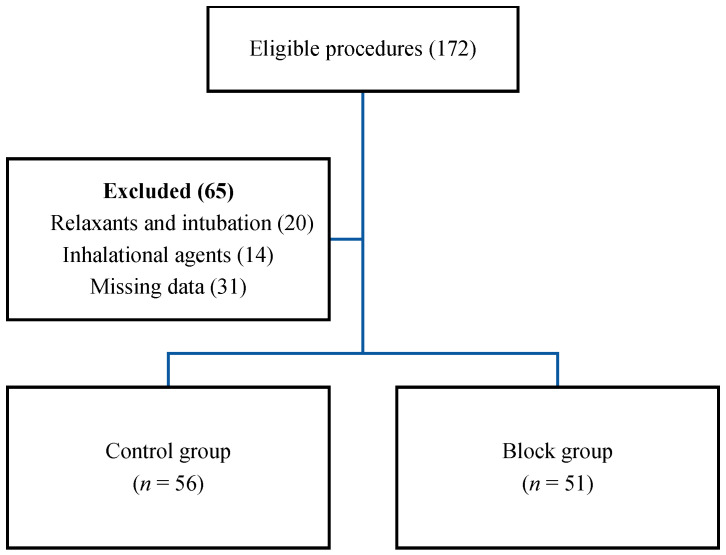
Screening, inclusion, and exclusion.

With the use of the surgery procedure codes, a total of 358 patients were found to be suitable for the study. After further screening, 172 patients met the inclusion criteria and became eligible for analysis. After further scrutiny of the records using the exclusion criteria, 65 patients were excluded due to the use of relaxants, need for intubation, and missing data (Figure 1). A total of 107 patients, 51 from the Block and 56 from the Control groups, were eligible for final analysis.

Demographic variables are summarized in Table 1. The groups were comparable with all demographic parameters. Important parameters such as age (mean 62 and 66 years in the Block and Control groups, respectively, *p* = 0.514), BMI (26 and 25, *p* = 0.257), and smoking (23.5% and 25%, respectively) showed no difference between the groups. In the preoperative period, the patients in the Block group received NSAIDs less often (*p* = 0.005), while no difference was found in the preoperative antiemetics (*p* = 0.184). The distribution of the American Society of Anesthesiology (ASA) physical status score was different between the groups, as the Block group has more ASA II and fewer ASA I patients than the Control group (*p* = 0.0006).

The median surgery time was significantly less in the Block group in comparison with the Control group (78 min (60–99) vs. 98.5 min (77.5–139.5); *p* = 0.005). Consequently, the median anesthesia time was also shorter in the Block group when compared with the Control group (140 min (115–166) vs. 160 min (132 to 188); *p* = 0.027). However, the time in PACU did not show a statistically significant difference (89 min (70–124) vs. 80 min (60–120); *p* = 0.196), while the total procedure time was lower in the Block group (224 min (151–255) vs. 264.4 min (211–323); *p* = 0.013) (Table 2).

The proportion of patients reporting pain on arrival (17.6% vs. 10.7%; *p* = 0.302 in the Block and Control groups, respectively) was not significantly different. Similarly, no difference was observed between the groups with respect to the incidence of nausea (0.5% vs. 7.1%; *p* = 0.052) in the Block and Control groups, respectively (Table 2).

Patients in the Control group received NSAIDs more frequently than the the patients in the Block group during the stay in the the PACU (26.8% vs. 5.9% (*p* = 0.004)).

Overall, patients in the Block group received statistically less opioids (converted to morphine) during the entire procedure period (anesthesia and PACU time combined) as compared with the Control group with the median of 16.4 mg vs. 31.2 mg (*p* < 0.0001) in the Block and Control groups, respectively. However, during PACU, the Block group patients consumed more opioids compared with the Control group patients (51.0% vs. 8.9%; *p* < 0.001). Further, the number of pain-free patients with a VRS (verbal rating score) of 0 was significantly lower in the Block group patients (51.0% vs. 80.4%, *p* = 0.001).

## 4. Discussion

In this retrospective comparative analysis of the data of breast cancer surgery patients, in which one group received the PECS II block as a supplement to general anesthesia, it was observed that surgery time and anesthesia time were reduced after the supplementation of the block. However, no influence on the PACU time was observed. Similarly, opioid consumption during the intraoperative period, and throughout the entire procedure, was significantly lower in patients receiving the block. In contrast to that, opioid consumption during the stay in the PACU was significantly higher in the block group. Further, no significant difference was seen in the incidences of pain and nausea between the groups.

The finding of shorter surgery and anesthesia time in the Block group may not be directly connected to the facilitation of the surgical procedure secondary to facial plane separation caused by administration of local anesthetic in between the muscles. However, one could speculate that hemodynamic stability achieved by better pain control may assist in reduced time used for hemostasis. Previous studies have suggested that higher intraoperative blood pressure variability is associated with increased blood loss during surgery [17], and peripheral nerve blocks may contribute to hemodynamic stability when combined with general anesthesia [18,19]. As the administration of additional opioids during surgery in both groups was directed by conventional parameters such as blood pressure and heart rate, reduced consumption in the Block group may reflect stable hemodynamics. This might provide a relatively bloodless surgical field hence less time for hemostasis. However, this is only speculation.

The observation of there being no significant difference in the incidence of pain on arrival in PACU in both groups, along with the requirement of intraoperative additional opioids and higher remifentanil infusion rates in Block patients, may reflect the inadequate efficacy of the PECS II block. This may be secondary to the possibility that the anterior cutaneous branches of the second to sixth thoracic intercostal nerves and branches of the supraclavicular nerves, which innervate the important parasternal surgical area of the breast, may not get blocked [14,15,20]. This may consequently create a demand for additional opioids intraoperatively and higher doses of analgesics at arrival in the PACU, especially when the analgesic effect of the remifentanil infusion is absent.

Further, the observation that differential consumption of analgesics exists, i.e., higher doses of morphine in the Block group and higher incidences of NSAIDs in the Control group during the PACU stay, warranted explanation. This may, although speculative, be the reflection of a general approach for analgesic therapy, where opioids are administered at higher VRS scores as it was in the Block group, while NSAIDs are preferred for lower VRS scores as it was in the Control group.

Another interesting observation was that both groups in the present analysis had low and statistically indifferent incidences of nausea, even though the Control patients received statistically significant higher doses of opioids. This was despite the fact that patients undergoing mastectomy with axillary dissection are at a particularly high risk of PONV with a reported incidence of 60–80%, especially when not receiving antiemetics [21,22].

Similar observations were documented in a recent meta-analysis of the efficacy of PECS block by B. Versyck et al., where the authors were unable to demonstrate a significant reduction in PONV to accompany the reduction in opioid consumption [23].

The direct antiemetic effect of propofol may be one of the explanations, as its use for the induction and maintenance of anesthesia has been shown to be associated with a lower incidence of PONV, as compared with any other anesthetic drug or technique [24].

Additionally, in a previous study on PONV in breast cancer patients, Bakshi SG et al. reported that the incidence of PONV is higher for patients below 50 years of age and that there is a positive association between estrogen receptor positivity and PONV in patients above 50 years of age, which may be attributed to the altered hormonal milieu in these patients [25]. In our study, as the average age of patients was above 60 years, lower incidences of PONV in both groups may be a secondary to the reformed hormonal milieu.

Further, the parameter of interest for the assessment of a logistical advantage of the block, i.e., the duration of stay in the PACU, showed no difference between the groups. This may be attributed to the fact that both the groups had similar incidences in parameters such as pain and nausea, which correlate with pain intensity, nausea, and vomiting on arrival [26].

The finding of similar incidences of PONV may be further supported by the fact that both the groups received opioids, i.e., the Control group during surgery and the Block group during their time in the PACU. which are major confounding factors for PONV [27].

The fact that both groups had a similar duration of stay during the full procedure, despite reduced surgery time in the Block group, and a similar length of time in the PACU, warrants further discussion. The utilization of the operation theaters depends on multiple factors, which include surgical time, anesthesia time, shifting time, the preparation of the operation theater for the next operation, the availability of beds and personnel in the PACU, etc. Thus, it is clear that, although the focus remains predominantly on surgery time, logistical challenges may significantly delay the use of operation theaters for the next patient.

This observation appears similar to previous studies, in which the influence of logistical factors on the PACU stay was emphasized [28,29,30]. The authors in the study used various fast-track anesthesia techniques for cardiac surgery patients. The concept of eligibility to discharge, which was based on the modified recovery room discharge score scheme, was used to accurately assess the influence of the different anesthesia regimens. Here, the authors found no advantage either in eligibility to discharge or in actual discharge from the PACU, despite implementation of the fast-tracking strategies. In our study, eligibility to discharge could not be assessed due to insufficient data.

Overall, as PACU occupancy influences operation theater utilization, a similar duration of stay in the PACU in both groups may not offer a logistical advantage in resource optimization, despite a significant reduction in surgery and anesthesia time, along with reduced intraoperative and total opioid consumption.

## 5. Limitations

Being a retrospective study, this analysis had several limitations. As the implementation of surgery, anesthesia, and its related documentation during the study period was not designed for future scrutiny, the data had significant variations which meant it was at times speculative. Sufficient hemodynamic data were unavailable in the records, which did not allow the authors to perform a meticulous analysis of positive or negative effects of the two-anesthesia regimen, fluid supplementations, requirement of vasopressors, etc.

Although the surgical protocol throughout the study period was unchanged, the influence of individual variations in the results should not be denied. Similarly, the effect of the learning curve of the PECSII block on the data may not be ignored.

The existence of factors such as perioperative anxiety and chronic pain conditions in patients can influence the consumption of analgesics in the postoperative period, irrespective of the actual severity of pain. Due to the retrospective nature of this study, the selection and randomization of such patients was not feasible.

The main aim of the study was to assess whether the PECSII block offers the possibility of acceleration in operation theater turnover, which is apparently dependent on PACU time. As logistical factors distort the PACU time data, more specific indicators such as eligible time to discharge may have offered more accurate results.

Finally, the retrospective design of the study can only indicate associations related to the effect of PECS block use.

## 6. Conclusions

The supplementation of the interpectoral-pectoserratus plane (PECS II) block reduces surgery and anesthesia time but does not influence PACU time. The PECS II block offers no advantage for resource optimization.

## Figures and Tables

**Table 1:** Pre-operative demographics: Total Propofol and Remifentanil consumption was similar in both the groups. However, by analyzing the consumption of Propofol over time in mg/kg/min, we found that patients in the Block group received marginally higher amounts of Propofol. In contrast, the use of Fentanyl and converted opioids was significantly lower in the Block group (Table 2). Thus, the Block group showed statistically lower opioid consumption during the intraoperative period with a median of 133 (80–233) µg in the Control group, as compared with 60 (33–100) µg in the Block group (*p* < 0.001).

Factor	Block Group	Control Group	*p*-Value
Number of patients	51	56	
Age (years)	62 (50–70)	66 (51–75)	0.514
Weight (kg)	71 (61–81)	70 (60–76)	0.160
BMI	26 (23–29)	25 (22–28)	0.257
Number of smokers (%)	12 (23.5)	14 (25.0)	0.859 *^)^
NSAID pre-OP (%)	2 (3.9)	20 (35.7)	0.005 *^)^
Antiemetic pre (%)	49 (96.1)	50 (89.3)	0.184 *^)^
ASA I (%)	5 (9.8)	19 (33.9)	
ASA II (%)	35 (68.6)	32 (57.4)	0.006 *^)^
ASA-score III (%)	11 (21.6%)	5 (8.9%)	

Data median (IQR) or number (%). Statistics: *) χ^2^ test, rest Mann–Whitney test. Abbreviations: BMI: body mass index. ASA: American Society of Anesthesiology. NSAID: non-steroidal anti-inflammatory drug

**Table 2:** Perioperative events and drugs.

	Block Group	Control Group	*p*-Value
*Peroperative*			
Anesthesia time (min)	140 (115–166)	160 (132–188)	0.027
Surgery time (min)	78.0 (60.0–99.0)	98.5 (77.5–139.5)	0.005
Total procedure time (min)	224 (171–255)	264 (211–323)	0.013
Propofol (mg/kg/min)	0.12 (0.10–0.15)	0.11 (0.09–0.13)	0.005
Remifentanil (mcg/kg/min)	0.37 (0.30–0.50)	0.38 (0.29–0.48)	0.629
Sufentanil/Fentanyl (μg)	60 (25–100)	125 (80–225)	<0.0001
Opioids corrected (mg)	60 (33–100)	133 (80–233)	<0.0001
Opioids to Morphine	12 (8–23)	27 (16–47)	0.0003
*Postoperative*			
Time in PACU (min)	89 (70–124)	80 (60–120)	0.196
VCR score 0	26 (51.0)	45 (80.4)	0.001
Nausea in PACU number (%)	1 (0.5)	4 (7.1)	0.052 *^)^
Pain at arrival number (%)	9 (17.6)	6 (10.7)	0.302 *^)^
NSAID in PACU number (%)	3 (5.9)	15 (26.8)	0.004 *^)^
Opioids number (%)	25 (51.0)	5 (8.9)	<0.0001
Opioids corrected (mg)	11.18 ± 16.7	1.29 ± 4.15	<0.0001 ^#)^
*Perioperative*			
Total opioids corrected	16.4 ± 12.9	31.2 ± 21.9	<0.0001 ^#)^

Abbreviations: PACU: Post-anesthesia care unit. NSAID: non-steroidal anti-inflammatory drug. Opioid conversion from National Drug and Alcohol Research Centre, University of NSW. Statistics: *) χ^2^ test, #) ANOVA one way, rest Kruskall–Wallis test. “https://ndarc.med.unsw.edu.au/sites/default/files/ndarc/resources/TR.329.pdf (accessed on 24 January 2023)”.

## Data Availability

The data were retrieved from various databases which can be accessed with appropriate permission and may take time.
